# Analyzing the demographics of patients with uveitis in an indigent, urban population

**DOI:** 10.1186/s12886-023-02888-3

**Published:** 2023-04-05

**Authors:** Caroline W. Tipton, Grace R. Reilly, Kevin Chen, Eileen Chang, Jessica M. Ackert, Paulina Liberman, Meghan K. Berkenstock

**Affiliations:** 1 Drexel University College of Medicine, Philadelphia, PA USA; 2 Massachusetts Eye and Ear Infirmary, Harvard Medical School, Boston, MA USA; 3 Departamento de Oftalmología, Escuela de Medicina. Pontificia Universidad Católica de Chile, Santiago, Chile; 4 Wilmer Eye Institute, Johns Hopkins School of Medicine, 600 N. Wolfe Street Maumenee 3rd Floor, Baltimore, MD 21087 USA

**Keywords:** Philadelphia, Uveitis, Indigent, Low-cost care

## Abstract

**Purpose:**

To study the types of uveitis examined in a hospital serving indigent populations in need of low-cost care.

**Methods:**

A retrospective chart review examined the electronic medical records of all patients with uveitis-related at Drexel Eye Physicians. Data collected included demographics, anatomic location of the uveitis, systemic disease associations, treatment modalities and insurance. Statistical analysis was performed using χ² or Fischer exact tests.

**Results:**

270 patients (366 eyes) were included for analysis, 67% of patients identified as African American. Most eyes (95.3%, N = 349) were treated with topical corticosteroid drops, and only 6 (1.6%) received an intravitreal implant. Immunosuppressive medications were started in 24 patients (8.9%). Nearly 80% depended to some extent on Medicare or Medicaid Assistance for treatment coverage. There was no association between insurance type and use of biologics or difluprednate.

**Conclusion:**

We found no association between insurance type and the prescription of medications for uveitis that should be used at home. There was a minimal number of patients prescribed medications for implantation in the office. The adherence of use of medications at home should be investigated.

## Introduction

Patients with uveitis require therapies ranging from corticosteroid eye drops to immunosuppressive agents to achieve quiescence of the inflammation [1, 2]. The side effects of these medications can vary and require recurrent lab monitoring and long-term ophthalmic follow-up [3]. Adding together the healthcare expenses associated with the medications, phlebotomy, and recurrent office visits, the long term management of uveitis can carry a high cost [4–6]. This may effectively eliminate more expensive drugs as treatment options for financially constrained patient populations [4–6]. One study assessed this cost using a large administrative claims database in the United States [7]. They found that in patients with the highest costs associated with treatment of non-infectious uveitis, the average annual total health care expenditure ranged from $59,873 to $349,967 during the 9 year study period [7]. In the Multicenter Uveitis and Steroid Treatment (MUST) trial, the combined cost of medications, surgeries, intravitreal implants, and hospital care was found to be $69,300 in patients receiving the fluocinolone implant for bilateral disease versus $52,500 for patients receiving systemic treatment with immunosuppressive agents [8].

Only one study has explored the impact of medication cost on adherence in patients with uveitis [9]. This study specifically looked at adherence barriers in a sample of pediatric patients. The literature on medication selection by the treating physician based on cost has disparate conclusions depending on the anatomic location of uveitis being treated, which ranges from no effect to strictly choosing therapy based on price [10–12]. Understanding that there are financial constraints to treatment and adherence in patients with uveitis is only half of the problem. Practically speaking, how do we limit the cost of eye drops and systemic immunosuppressive agents to provide affordable treatments which promote adherence in patients with chronic uveitis?

Currently, there are no studies that identified strategies to limit healthcare costs associated with medications in indigent populations requiring low-cost care for the treatment of uveitis. Drexel Eye Physicians (DEP) was an academic ophthalmology practice affiliated with the safety net hospital, Hahnemann University Hospital (HUH). HUH served the primarily uninsured, Medicaid, and Medicare population in Central Philadelphia [13, 14]. With operating costs outstripping the reimbursement rate, Hahnemann University Hospital had amassed a monthly loss of 3–5 million dollars and was forced to file for bankruptcy protection [14].

Prior to the closure of HUH and the DEP in September 2019, we examined the uveitides seen to understand the treatments needed for control of ocular inflammation. We concurrently analyzed patient insurance plans and eye drop copays to assess prescribing practices used to decrease treatment cost.

## Methods

A retrospective chart review of the paper and electronic medical records of 270 patients (366 eyes) with uveitis-related ICD-9 codes was conducted from September 1, 2011 through September 30, 2014 at the Drexel Eye Physicians practice, Philadelphia, PA. Demographic information collected included sex, race, laterality, uveitis and glaucoma treatments, uveitis diagnosis, chronicity, anatomic location, and medical comorbidities (including systemic autoimmune diseases). Uveitis treatments consisted of corticosteroid eye drops, intra- and peri-ocular corticosteroid injections, and systemic immunosuppressive agents. Glaucoma type, eye drop use, and need for surgical intervention were recorded. At the time of data collection, paper charts were being scanned for uploading into the new electronic medical record and subsequently sent to a secured storage facility. Therefore, only 155 of the 270 charts were available to record insurance information, including primary and secondary plans. Copay costs for generic drugs, including eye drops, were obtained from information on insurance plan formularies available to office staff at the time of data collection.

The data was collected using Excel software (Microsoft Corporation, Redmond, WA), and analyzed using IBM SPSS Statistics for Windows, version 22 (IBM Corp., Armonk, N.Y., USA). The interrelationships between categorical variables of interest, and their statistical significance, were explored using χ [2] or Fisher’s exact tests, where appropriate. A p-value of ≤ 0.05 was considered significant. The study was approved by the Drexel University College of Medicine Institutional Review Board and followed the tenets of the Declaration of Helsinki.

## Results

A total of 270 patients (366 eyes) were included for analysis and their demographic information is outlined in Table 1. The majority of patients were female (61.5%, N = 166) and the most common racial group was African-Americans (67%, N = 181) followed by Caucasians (16.3%, N = 44). Only 20% of patients (N = 54) were diagnosed with an underlying systemic autoimmune disease, of which sarcoidosis was the most common (25%, N = 14).

**Table 1:** Patient demographics including sex, race, and systemic disease associations

N = 270 patients	No. (%)	
**Sex**		
Female	166 (61.5)	
**Race/Ethnicity**		
African-American	181 (67.0)	
Caucasian	44 (16.3)	
Hispanic	26 (9.6)	
Asian	11 (4.1)	
Middle-Eastern	7 (2.6)	
Unknown	1 (0.4)	
**Systemic Disease Associations**	184 (68.1)	
Diabetes Mellitus	68 (25.2)	
Hypertension	112 (41.5)	
HIV	7 (2.5)	
Renal Transplant	2 (0.7)	
**Systemic Autoimmune Diseases (N = 54)**		
Sarcoidosis	14 (25.0)	
Rheumatoid Arthritis	6 (11.1)	
Crohn’s Disease	5 (9.3)	
Mixed Connective Tissue Disease	4 (7.6)	
Behcet’s Disease	3 (5.6)	
HLA-B27 Spondyloarthropathy	3 (5.6)	
Systemic Lupus Erythematosus	3 (5.6)	
Ulcerative Colitis	3 (5.6)	
Juvenile Idiopathic Arthritis	2 (3.7)	
Lambert-Eaton Syndrome	1 (1.9)	
Multiple Sclerosis	1 (1.9)	
Pemphigoid	1 (1.9)	
Sjogren Syndrome	1 (1.9)	
Scleroderma	1 (1.9)	
Other Autoimmune Disease*	1 (1.9)	

*SLE = systemic lupus erythematosus, HIV = human immunodeficiency virus*

*Includes an overlap syndrome of rheumatoid arthritis, Sjogren’s syndrome, sarcoidosis, SLE

Table 2 details the laterality and chronicity of the uveitides diagnosed within the study population. Most patients had unilateral disease (64.4%) and the most common anatomic location of uveitis was anterior (75.6%), of which 51.3% were acute in onset. Of note, post-operative anterior chamber inflammation comprised 17.3% of all anterior uveitis cases. However, analysis of the relationships between race and uveitis secondary to post-operative inflammation did not produce statistically significant results. Overall, 15.6% (N = 57) were diagnosed with glaucoma or being followed as a glaucoma suspect, as shown in Table 2. Of those, 63.2% (N = 36) were African American, and 36.8% (N = 21) were Caucasian. The most common type of glaucoma was primary open angle glaucoma (POAG) or POAG suspect, of which 70% (N = 27) were African American. All patients with combined mechanism, chronic angle closure, neovascular, steroid-induced glaucoma, or anatomically narrow angles identified as African American. A detailed list of the specific uveitis diagnoses is noted in Table 3.

**Table 2:** Ocular characteristics including laterality, chronicity, and location of uveitis

N = 366 eyes	No. (%)
**Laterality**	
Bilateral	192 (52.5)
**Anatomic Location of Uveitis**	
Anterior	277 (75.5)
• Acute	142 (38.8)
• Recurrent Acute	21 (5.7)
• Chronic	114 (31.1)
Intermediate	5 (1.4)
Posterior	19 (5.2)
Panuveitis	58 (15.8)
Sclerouveitis	7 (1.9)
**Glaucoma or Glaucoma-Suspect**	57 (15.6)

**Table 3:** Causes of uveitis by anatomic location

N = 366 eyes	No. (% of each category)
**Anterior uveitis**	
Post-operative, Non-infectious	48 (17.3)
Traumatic	23 (8.3)
Sarcoidosis	13 (4.7)
Varicella Zoster	8 (2.8)
HLA-B27 Associated	6 (2.2)
Herpes Simplex	5 (1.8)
Juvenile Idiopathic Arthritis	4 (1.4)
Syphilis	2 (0.7)
Fuchs Uveitis Syndrome	1 (0.4)
Post-operative, *P. acnes* Infection	1 (0.4)
Idiopathic	166 (59.9)
**Intermediate Uveitis**	
Sarcoidosis Associated	2 (40)
Idiopathic	3 (60)
**Posterior Uveitis**	
Toxoplasmosis Retinitis	5 (26.3)
Bartonella neuroretinitis	3 (15.8)
Relentless Placoid Chorioretinitis	2 (10.5)
Acute Retinal Necrosis	1 (5.3)
Cytomegalovirus Retinitis	1 (5.3)
Serpiginous Choroiditis	1 (5.3)
Idiopathic	6 (31.6)
**Panuveitis**	
Sarcoidosis	12 (20.7)
Behcet’s Disease	6 (10.3)
Multifocal Choroiditis and Panuveitis	4 (6.9)
Vogt-Koyanagi-Harada Disease	4 (6.9)
Post-operative Endophthalmitis	2 (3.4)
Multiple Sclerosis	1 (1.7)
Idiopathic	29 (50%)

While most eyes (95.3%) were treated with topical corticosteroid drops, 22 eyes (6%) received periocular or intravitreal corticosteroid injections individually or both sequentially during the study; immunosuppressive medications were started in 24 patients (8.9%) (Table 4).

**Table 4:** Uveitis treatment type by eye and by patient including drops, periocular and intravitreal injections, and systemic treatments

N = 366 eyes	No. (%)
**Corticosteroid Drops** Prednisolone Acetate 1% Difluprednate 0.05%	349 (95.3) 291 (79.5) 58 (15.8)
**Regional Steroid Injection(s)** Sub-tenon Triamcinolone Sub-tenon Triamcinolone then IVTA IVTA then Dexamethasone 0.7 mg implant Dexamethasone 0.7 mg implant	22 (6) 13 (3.6) 3 (0.8) 3 (0.8) 3 (0.8)
**Antimicrobial treatment (eye level)**	
Topical ganciclovir	3 (0.8)
Intravitreal antibiotics	2 (0.5)
Trifluridine	1 (0.3)
Ofloxacin	1 (0.3)
**Glaucoma Drops**	43 (11.7)
Glaucoma Surgery or Lasers* **Laser peripheral iridotomy (LPI)** **Trabeculectomy** **Tube**	9 (2.5) 2 (0.5) 6 (1.6) 3 (0.8)
**N = 270 patients**	
**Systemic Prednisone**	44 (16.3)
**Antimicrobial treatment (person level)**	
Valacyclovir	7 (2.6)
Acyclovir	3 (1.1)
Famvir	3 (1.1)
Topical ganciclovir	3 (1.1)
Trimethoprim sulfamethoxazole	3 (1.1)
Valganciclovir	1 (0.4)
Intravenous antibiotics	1 (0.4)
**Immunosuppressive Agents Overall**	24 (8.9)
**Antimetabolites** • Methotrexate • Mycophenolate mofetil • Azathioprine	18 (6.7) 13 3 2
**Biologic Agents** • Infliximab • Adalimumab • Abatacept	11 (4.1) 7 3 1

IVTA = Intravitreal triamcinolone acetonide; * two eyes required multiple procedures: one eye had an LPI and trabeculectomy, one had a tube and trabeculectomy

Of the 24 patients who started corticosteroid sparing immunosuppressive therapies, most (68.2%, N = 16) were treated concurrently with a 3-month prednisone taper versus intravitreal triamcinolone (N = 2) to control inflammation until the immunosuppressive agent was fully effective. Antimetabolites were prescribed for 18 patients. Only 11 patients required subsequent use of biologic agents for uveitis treatment.

The relationship between the use of immunosuppressive agents and laterality of uveitis diagnosis was analyzed, with bilateral disease significantly more likely to be treated with immunosuppressive agents (*p* < 0.001). The relationship between use of immunosuppressive agents with posterior pole involvement (panuveitis or posterior uveitis) and concurrent autoimmune disease were both statistically significant, p < 0.001 and *p* = 0.001, respectively. Of the 11 patients on biologic agents, 8 (72%) had a systemic autoimmune disorder requiring treatment.

Of the 270 patients who underwent treatment for uveitis, 115 (42.6%) of patients had their insurance type recorded. Fifty-six patients (48.7%) subscribed to one insurance plan, while 59 patients (51.3%) received both primary and secondary coverage. The most frequent insurance type used was the combination of a Medicare primary plan with Medicaid as the secondary supplement (29.6%). The majority (80%) of patients required use of Medicare or Medicaid insurance. Only 1 patient was uninsured. A review of formulary costs from these plans, showed a range of out-of-pocket copays ranging from $0–80 dollars per eye drop bottle, with a median of $4. The average retail price of prednisolone acetate 1% at the time of the study without insurance coverage was $50.

The relationship between insurance type and uveitis treatment was assessed. The relationship between use of biologic agents and insurance type (Table 5) was not statistically significant (*p* = 0.582) overall or when specifically assessed in patients with commercial insurance plans (*p* = 0.299). Similarly, the relationship between difluprednate use and insurance type was not statistically significant overall (*p* = 0.840) or with commercial insurance coverage (*p* = 0.930).

**Table 5:** Number of insurance plans and types utilized for treatment coverage

N = 115 Patients	No. (%)
Number of Insurance Plan(s)	
One	56 (48.7)
Two	59 (51.3)
**Insurance Type (Primary +/- Secondary)**	
Medicare + Medicaid	34 (29.6)
Medicaid	24 (20.9)
Commercial	21 (18.3)
Medicare + Commercial	17 (14.8)
Medicare	10 (8.7)
Medicaid + State sponsored, non-Medicaid secondary	4 (3.5)
Medicaid + Commercial	3 (2.6)
Commercial primary and secondary	1 (0.9)
Uninsured	1 (0.9)

## Discussion

Treatment of uveitis requires suppression of the ocular inflammation to decrease the development of secondary complications while preventing blindness and disability over the long term [15]. Ultimately, the success of treatment is reliant upon patient adherence and the ability to maintain the regimen over time. An important, and previously unstudied, factor in treatment adherence is the patient’s ability to afford the cost of treatment. While anterior uveitis accounts for approximately 50–90% of uveitis cases, posterior and panuveitis have higher associated medical costs and rates of blindness [15, 16]. Moreover, chronic uveitis requiring long-term use of immunosuppressive agents further contributes to a higher cost of treatment, especially when factoring in the cost of phlebotomy and treatment side effects associated with use [15]. Therefore, chronic, posterior pole involving uveitides are at the highest risk for treatment non-adherence and poor visual outcomes, which is a valid concern when treating indigent populations with limited medical coverage.

A prominent feature of our study is the high prevalence of African American patients (67%), which is slightly higher than the percent of African Americans in the Philadelphia population in the 2010 census (41%).^17^ Our cohort demonstrated more cases of sarcoidosis compared to previously published studies where the predominant underlying autoimmune disorder was HLA-B27 associated [18, 19]. As the incidence of sarcoidosis is higher in African Americans than Caucasians in the United States, and it follows that the patients in our study would have a higher prevalence of sarcoid uveitis [20]. Another point of difference compared to prior studies was the larger percentage of post-operative anterior uveitis (17.3% of anterior cases, 13% overall). However, post-operative inflammation was not found to be statistically more common in a one specific race despite previous studies citing African American race as a risk factor for persistent post-operative iritis [21]. Interestingly, 24 of the 48 eyes with post-operative inflammation were in patients with diabetes mellitus. There has been an association with the development of uveitis in diabetics; specifically, poor glycemic control was posited as a cause for uveitis activation [22].

Given most patients in the study were diagnosed with non-infectious inflammation involving the anterior chamber, a cost-effective option was the use of prednisolone acetate 1%. In a more recent study from 2019, anti-inflammatory eye drop out-of-pocket prices were compared between pharmacies in the United States, and ranged for prednisolone acetate 1% from $48.82 to $51.61 [23]. Comparatively, difluprednate was priced in the $211.36 to $216.85 range [23]. Copays for difluprednate ranged widely given variable formulary coverage, which led to providers learning which medications were covered by a patient’s insurance plan to tailor prescribing practices on an individual basis. Through the prescription of formulary and generic eye drops, the median copay in for all pharmacologic classes of eye drops in this study was kept exceptionally low at $4.

In those with chronic anterior uveitis or with posterior involvement, prednisone was given during induction of immunosuppressive therapy in the majority of patients for several reasons. It is readily available, generic, and is both effective and cost-effective until the immunosuppressive agent can reach full efficacy [15, 23]. Intravitreal steroid injections have recently been explored as a replacement to systemic steroid therapy due to their improved penetration [24, 25], However, studies have shown that the dexamethasone implant is associated with higher up-front costs, more frequent follow-ups, and more adverse effects, such as glaucoma and cataracts, compared to systemic steroids [26, 27]. A recent review found that the dexamethasone implant added an estimated £19,509 ($26,683 in 2021 American Dollars) per additional year of life [27]. In keeping with formulary medications and attempting to keep the treatment cost as low as possible, the dexamethasone pellet was implanted in just 6 (1.6%) eyes in this study. Unfortunately, with the low number of dexamethasone implants used, a statistical analysis of the type of insurance plan and the use of the implants could not be assessed.

After initial therapy with oral or regional corticosteroids, our results mirrored prior studies where nearly two-thirds of patients have been shown to have resolution of their disease within 2 years on a single immunosuppressant, 20–25% of patients require an additional drug for symptom resolution. Only the minority of patients (N = 5, 21%) required more than one immunosuppressant for the control of their ocular inflammation, of which 75% were initially on antimetabolites. In our cohort, biologic agents were selectively used in patients requiring it for systemic disease or used as a second-line agent in patients requiring escalation in therapy to control ocular inflammation. Previous publications have shown that this prescribing practice has been shown to defray treatment costs as patients with the highest healthcare spending were more likely to have a diagnosed autoimmune condition and require biologic medications [7].

For those patients requiring use of biologic medications, the cost of treatment was defrayed through several mechanisms, which included subscription to pharmaceutical company sponsored financial support programs for those with commercial insurance plans. In the case of adalimumab, it was found that joining patient support program helped lower the rate of medication discontinuation and resulted in a statistically significant lower overall disease cost despite higher individual drug cost [28]. As previously mentioned, the majority of patients treated with biologic agents had an underlying rheumatologic condition. Obtaining a prior authorization through use of the ICD-9 code for the systemic disorder facilitated insurance approval in all cases, as the biologic agent was FDA approved for use for the rheumatologic condition versus being used off label for the sole treatment of the uveitis. By partnering with the Drexel University College of Medicine rheumatology division to undertake the prior authorization and appeals process, patients received streamlined medical care with a decreased cost.

It is important to note that our study does have several limitations due to its retrospective nature and relatively small number of patients. Similarly, treatment was not standardized and was at the discretion of the treating physician. However, the data do not show that more expensive drugs were prescribed to patients with private insurance and less costly to those with government-sponsored plans. Implementation of preferred practice patterns and prompt referral to the uveitis specialist upon diagnosis were only starting to be implemented at the end of the study period. The effect of all providers referring to a uveitis trained subspecialist on the streamlining of laboratory evaluations, referrals to rheumatology, and clinical outcomes were not assessed. Further follow-up was needed to determine the effect of these cost saving mechanisms on a person-year and eye-year basis as well as the rate of medication adherence. However, the closure of HUH and the DEP practice precluded these analyses. Without medication adherence data and correlated patient outcomes, we cannot draw conclusions on treatment effectiveness.

In summary, our study examined a diverse group of patients from lower socioeconomic backgrounds, 80% of patients with recorded insurance depended to some extent on federally funded insurance programs for treatment coverage. This study provides practical tips to lower drug and overall treatment costs in patients with uveitis. Further studies are needed to asses different aspects of barriers to care. The relation between adherence of use of medications at home and insurance type should be specifically addressed in those studies.

## Data Availability

The datasets used and analyzed during the current study are available from the corresponding author on reasonable request.
